# Filogenia y resistencia de cepas de *Escherichia coli* productoras de betalactamasas de espectro extendido a los antibióticos en pacientes con cáncer hospitalizados en Perú

**DOI:** 10.7705/biomedica.6263

**Published:** 2022-09-02

**Authors:** José Matta-Chuquisapon, Esther Valencia-Bazalar, Carlos Sevilla-Andrade, Helí Jaime Barrón-Pastor

**Affiliations:** 1 Grupo de Investigación Resistencia a los Antimicrobianos, Universidad Nacional Mayor de San Marcos, Lima, Perú Universidad Nacional Mayor de San Marcos Grupo de Investigación Resistencia a los Antimicrobianos Universidad Nacional Mayor de San Marcos Lima Peru; 2 Centro de Investigaciones Tecnológicas, Biomédicas y Medioambientales, Universidad Nacional Mayor de San Marcos, Lima, Perú Universidad Nacional Mayor de San Marcos Centro de Investigaciones Tecnológicas, Biomédicas y Medioambientales Universidad Nacional Mayor de San Marcos Lima Peru; 3 Centro de Investigación de Bioquímica y Nutrición “Alberto Guzmán Barrón”, Universidad Nacional Mayor de San Marcos, Lima, Perú Universidad Nacional Mayor de San Marcos Centro de Investigación de Bioquímica y Nutrición “Alberto Guzmán Barrón” Universidad Nacional Mayor de San Marcos Lima Peru

**Keywords:** Escherichia coli, filogenia, resistencia a betalactámicos, ciprofloxacina, gentamicinas, Escherichia coli, phylogeny, beta-lactam resistance, ciprofloxacin, gentamicins

## Abstract

**Introducción.:**

Las infecciones asociadas con la atención en salud constituyen un problema de salud pública porque aumentan la morbimortalidad de los pacientes, sobre todo de aquellos con factores de riesgo, como la inmunosupresión debida a enfermedades oncológicas. Es importante conocer la diversidad genética de los principales microorganimos causantes de infecciones hospitalarias mediante la vigilancia epidemiológica tradicional y la epidemiología molecular, para hacer un mejor seguimiento y detectar brotes tempranamente.

**Objetivo.:**

Determinar el grupo filogenético y la resistencia a antibióticos de las cepas de *Escherichia coli* aisladas de pacientes con cáncer hospitalizados.

**Materiales y métodos.:**

Se hizo un estudio de tipo transversal que incluyó 67 cepas de *Escherichia coli* productoras de betalactamasas de espectro extendido (BLEE). Se determinó el grupo filogenético, el perfil de resistencia a los antibióticos, los genes de resistencia a betalactámicos, el tipo de las muestras y los servicios de hospitalización de donde fueron recuperadas.

**Resultados.:**

El grupo filogenético más frecuente fue el B2 (36 %). El 57 % de las cepas B2 fueron aisladas de muestras de orina y el 33 % provenía del servicio de urología. La resistencia a ciprofloxacino y gentamicina fue de 91 y 53 %, respectivamente, y el 79 % de las cepas tenía el gen _*bla*_ *CTX-M*. Se encontró una relación significativa (p<0,05) entre los grupos filogenéticos y la resistencia a ciprofloxacina, así como a la edad del paciente.

**Conclusión.:**

El filogrupo de *E. coli* predominante fue el B2. Se evidenció una gran resistencia a ciprofloxacina y gentamicina, una proporción elevada de cepas BLEE con el _*bla*_ *CTX-M*, y una relación entre el grupo filogenético y la resistencia a ciprofloxacino.

## Abstract (en)

**Introduction:**

Healthcare-associated infections are a public health problem due to the increased morbimortality of patients, especially those with risk factors such as immunosuppression due to oncological diseases. It is essential to determine the genetic diversity of the main microorganisms causing healthcare infections by combining traditional epidemiological surveillance and molecular epidemiology for better outbreak follow-up and early detection.

**Objective:**

To determine the phylogenetic group and antibiotic resistance of *Escherichia coli* isolated from hospitalized oncologic patients.

**Materials and methods:**

We conducted a cross-sectional study of 67 strains of ESBL- producing *Escherichia coli* to determine their phylogenetic group and described their antibiotic resistance profile, beta-lactam resistance genes, as well as the type of sample and the hospitalization areas from which they were recovered.

**Results:**

The most frequent phylogenetic group was B2 (36%); 57% of B2 strains were isolated from urine and 33% came from the urology department. Resistance to ciprofloxacin and gentamicin was 92% and 53%, respectively, and 79% of the strains had the _*bla*_ *CTX-M* gene. A significant association (p<0.05) was found between the phylogenetic groups, ciprofloxacin resistance, and the age of the patients.

**Conclusion:**

The predominant *E. coli* phylogroup was B2. We evidenced high resistance to ciprofloxacin and gentamicin, a high proportion of ESBL strains with the _*bla*_ *CTX-M* gene, and a significant association between the phylogenetic group and the resistance to ciprofloxacin.

Las infecciones que se adquieren en los establecimientos de salud al cabo, por lo menos, de 48 horas del ingreso, se denominan infecciones asociadas con la atención en salud (IAAS) ^1^. Estas aumentan la morbimortalidad de los pacientes y los costos de la atención, y en algunos casos pueden tener implicaciones legales ^2^. Para comprender mejor cómo se generan y diseminan estas infecciones intrahospitalarias, se utilizan cada vez más las técnicas de epidemiología molecular enfocadas en las características genéticas del microorganismo (factores de virulencia, genes de resistencia a antibióticos, filogenia) y las características del huésped (grupo racial, sexo, estado inmunológico) ^3^. Los pacientes con cáncer son uno de los grupos con mayor riesgo de IAAS debido a su condición inmunológica (inmunosupresión) ^4^, por lo que los programas para prevenirlas incluyen el manejo para reducir el riesgo de infecciones en ellos ^5^.

En los pacientes con cáncer, además de verse afectados por las infecciones fúngicas, *Escherichia coli* es una de los principales bacterias causantes de IAAS ^5^, con el 30 % de los casos de bacteriemias; además, estos microorganismos frecuentemente son portadores de betalactamasas de espectro extendido (BLEE). Se han clasificado siete grupos filogenéticos (A, B1, B2, C, D, E, F) de *E. coli*^6^ mediante PCR múltiple, con resultados comparables a los de técnicas de mayor resolución como la tipificación multilocus de secuencias (*Multilocus Sequence Typing,* MLST).

Las cepas de *E. coli* que pertenecen a los grupos B2 y D, conocidas como patógenas extraintestinales, poseen factores de virulencia (sideróforos, adhesinas, exotoxinas, etc.) que les permiten sobrevivir fuera del intestino, así como mecanismos de resistencia a múltiples familias de antibióticos, entre ellas, los betalactámicos y las quinolonas ^7^. Cabe destacar que hay una cepa característica del grupo filogenético B2, la tipo ST131, que posee una betalactamasa CTX-M y una acetilasa acc-6,’ lo que le confiere resistencia a las quinolonas, y causa tanto infecciones de las vías urinarias como de los tejidos blandos ^8^.

El objetivo de este estudio fue describir la diversidad genética de los aislamientos clínicos de *E. coli*, determinando los grupos filogenéticos mediante PCR en pacientes con cáncer hospitalizados, la relación con el tipo de muestra, las áreas de hospitalización donde se encontraron y los niveles de resistencia a los antibióticos no betalactámicos y betalactámicos de amplio espectro como los carbapenémicos.

## Materiales y métodos

### Diseño y lugar de estudio

En este estudio de tipo transversal, se analizaron 67 cepas de *E. coli* productoras de betalactamasas de espectro extendido (BLEE) en el marco del proyecto “Vigilancia epidemiológica de bacterias resistentes en infecciones asociadas a la atención en salud en los servicios de hospitalización del Instituto Nacional de Enfermedades Neoplásicas (INEN), 2017-2018”.

Las cepas incluidas en el estudio se recolectaron durante el 2017 y provenían de diferentes muestras clínicas (orina, sangre, secreción bronquial) de pacientes hospitalizados. Los estudios moleculares se desarrollaron en el Laboratorio de Epidemiología Molecular y Genética (LEMYG) del Centro de Investigaciones Tecnológicas, Biomédicas y Medioambientales (CITBM) de la Universidad Nacional Mayor de San Marcos en Lima.

### Reactivación de aislamientos y extracción de ADN

La determinación de los grupos filogenéticos requería reactivar los aislamientos y extraer ADN de alta calidad y pureza. Los aislamientos, que estaban conservados a -80 ºC, se reactivaron en tres etapas:

- siembra en agar cromogénico-enterobacterias para evaluar la pureza;
- resiembra en agar tripticasa de soya para aislar colonias puras en un medio sin inhibidores, y
- inoculación en caldo Luria para empezar el protocolo de extracción de ADN con el estuche BactozolTM (Molecular Research Center, Inc.). Cada paso de resiembra antes de la extracción de ADN tuvo un periodo de incubación de 24 horas a 37 ºC.

### Determinación del grupo filogenético

**Figura 1:**
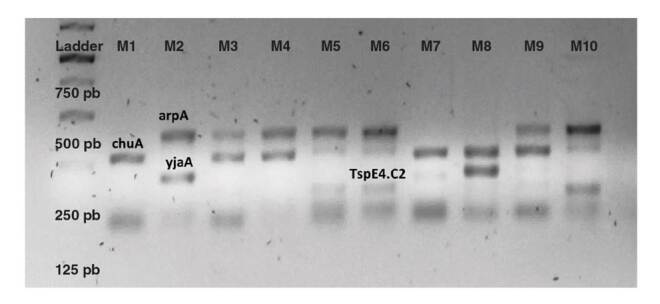
PCR múltiple para la determinación de los grupos filogenéticos de *Escherichia coli*. Se observan las cuatro bandas correspondientes a los tres iniciadores y un fragmento de ADN utilizados en el protocolo según Clermont, *et al*.

Los grupos filogenéticos se determinaron según lo descrito por Clermont, *et al*. ^6^. Los productos amplificados de la PCR múltiple se evaluaron mediante electroforesis en gel de agarosa al 2 %, posteriormente se tiñeron con el colorante RedSafe^TM^ y se observaron en un transiluminador de luz ultravioleta (figura 1).

### Sensibilidad a los antibióticos y genes de resistencia

Los resultados del estudio fenotípico de la sensibilidad a los antibióticos se expresaron como la concentración inhibitoria mínima (CIM) de cada familia de antibióticos utilizada. Esta concentración se calculó utilizando el equipo automatizado BD Phoenix^™^. Las categorías de sensibilidad antimicrobiana se establecieron con base en el manual CLSI M100 Ed 27 ^9^. El estudio molecular de la resistencia a betalactámicos se centró en determinar la presencia de los genes _*bla*_ *CTX-M,*_*bla*_ *KPC* y _*bla*_ *NDM*, utilizando los iniciadores descritos por Ghasemi, *et al*., ^10^ y las condiciones de PCR descritas por Chávez-Hidalgo ^11^. Se excluyeron del análisis los antibióticos betalactámicos afectados por las BLEE.

### Análisis estadístico

Se utilizaron la mediana y el rango intercuartílico (RIC) para las variables cuantitativas y, las proporciones, para las cualitativas. Con el test exacto de Fisher se estudiaron las asociaciones entre las variables categóricas de interés y la prueba U de Mann-Whitney o la de Kruskal-Wallis para las variables cuantitativas agrupadas por variable categórica.

### Aspectos éticos

El presente estudio contó con la aprobación del Comité Institucional de Ética en Investigación del Instituto de Medicina Tropical “Daniel Alcides Carrión” de la Universidad Nacional Mayor de San Marcos y con la autorización para el uso de datos de los investigadores del proyecto principal. El desarrollo del estudio siguió las directrices éticas y de buenas prácticas en investigación biomédica. Se analizaron aislamientos bacterianos que no fueron relacionados con el nombre del paciente ni la historia clínica.

## Resultados

### Características generales de los aislamientos

Los 67 aislamientos de *E. coli* provenían de pacientes con cáncer hospitalizados, con una mediana de edad de 55 años (RIC=28-71); el 67 % se habían obtenido en mujeres y las muestras más frecuentes eran de orina y sangre (54 y 42 %, respectivamente). Los servicios de hospitalización con la mayor cantidad de aislamientos recuperados fueron medicina interna y urología, con 30 % y 28 %, respectivamente (cuadro 1).

**Cuadro 1:** Distribución de los grupos filogenéticos de *Escherichia coli* y las características generales de los aislamientos

		Grupos filogenéticosa							
		Nb (%)	A	A o C	B1	B2	D o E	F	p^c^
			n (%)	n (%)	n (%)	n (%)	n (%)	n (%)	
Edad*		55 (28-71)	39 (32-46)	58 (29-74)	18 (4-42)	60 (53-75)	46 (18-64)	67 (55-80)	0,039
Sexo									
	Mujer	45 (67)	1 (50)	8 (57)	8 (80)	14 (67)	4 (67)	6 (100)	-
	Hombre	22 (33)	1 (50)	6 (43)	2 (20)	7 (33)	2 (33)	-	
Tipo de muestra									
	Secreción bronquial	3 (4,5)	-	-	-	2 (9,5)	-	1 (17)	-
	Orina	36 (54)	1 (50)	7 (50)	4 (40)	12 (57)	3 (50)	4 (67)	
	Sangre	28 (42)	1 (50)	7 (50)	6 (60)	7 (33)	3 (50)	1 (17)	
Servicio									
	Abdomen	6 (9)	-	2 (14)	-	3 (14)	-	1 (17)	-
	Cabeza y cuello	1 (1,5)	-	-	-	1 (4,8)	-	-	
	Ginecología	10 (15)	1 (50)	2 (14)	2 (20)	3 (14)	-	1 (17)	
	Mamas y tejidos blandos	1 (1,5)	-	-	-	-	1 (17)	-	
	Medicina interna	20 (30)	-	5 (36)	4 (40)	5 (24)	2 (33)	2 (33)	
	Pediatría	7 (10)	-	-	2 (20)	2 (9,5)	1 (17)	-	
	Tórax	1 (1.5)	-	-	-	-	-	1 (17)	
	UCI^d^	1 (1.5)	-	-	1 (10)	-	-	-	
	Urología	19 (28)	1 (50)	5 (36)	1 (10)	7 (33)	2 (33)	1 (17)	
Resistencia a los antibióticos									
	Gentamicina (R)	34 (51)	1 (50)	7 (50)	4 (40)	9 (43)	4 (67)	6 (100)	-
	Amikacina (R)	2 (3)	-	2 (14)	-	-	-	-	-
	Ciprofloxacina (R)	61 (91)	1 (50)	14 (100)	7 (70)	21 (100)	6 (100)	6 (100)	0,019
	Meropenem (R)	2 (3)	-	-	-	1 (4,8)	-	-	-
	Imipenem (R)	2 (3)	-	1 (7,1)	-	-	1 (17)	-	-

R: resistente

^*^Edad: mediana (RIC)

^a^8 aislamientos no fueron recuperados por lo que no se determinó su grupo filogenético.

^b^resultados para las 67 cepas

^c^prueba de Kruskal-Wallis; prueba de Fisher

^d^unidad de cuidados intensivos

### Resistencia a los antibióticos y grupos filogenéticos

**Figura 2:**
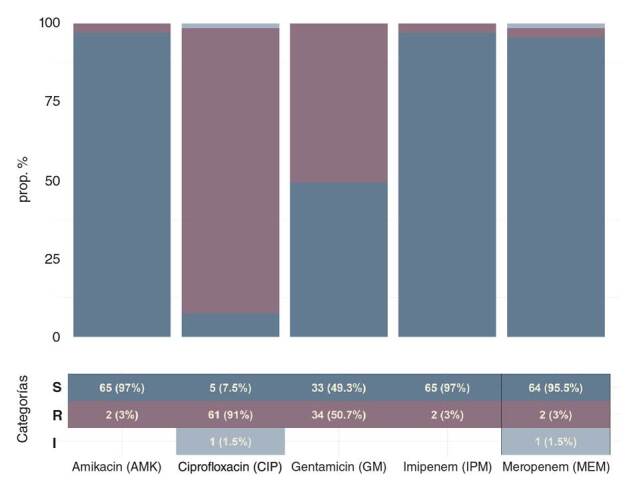
Perfil de resistencia a los antibióticos de las cepas aisladas de *Escherichia coli*

Se detectaron dos cepas de *E. coli* resistentes a carbapenémicos (3 % a meropenem y 3 % a imipenem). El 91 % (n=61) de las cepas era resistente a ciprofloxacina. En el caso de los aminoglucósidos, la amikacina mostró niveles bajos de resistencia, con un 3 % (n=2) de las cepas, pero la gentamicina registró hasta un 51 % (n=34) (figura 2). En el caso de los genes de resistencia, en el 79 % (n=53) de las cepas se encontraron betalactamasas de tipo _*bla*_ *CTX-M* (cuadro 2), y en dos se detectó resistencia enzimática a carbapenémicos (_*bla*_ *KPC* y _*bla*_ *NDM*).

**Cuadro 2:** Relación entre los antibióticos y las cepas CTX-M de *E. coli*

	CTX-M	No CTX-M	p ^a^
	n (%)	n (%)	
Gentamicina (R)	24 (45)	10 (71)	0,082
Amikacina (R)	2 (3,8)	-	-
Ciprofloxacino (R)	51 (96)	10 (79)	0,058
Meropenem (R)	-	2 (14)	0,041
Imipenem (R)	-	2 (14)	0,041

R: resistente

^a^ prueba de Fisher

**Figura 3:**
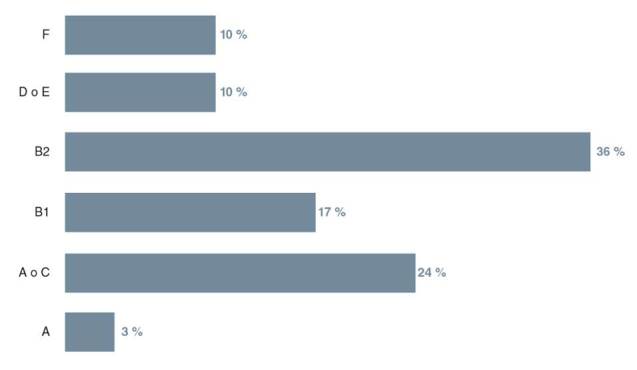
Porcentajes de los filogrupos determinados por PCR múltiple en los aislamientos de *Escherichia coli*

En cuanto a los grupos filogenéticos, 8 aislamientos de los 67 no pudieron ser reactivados para determinar su grupo filogenético. El grupo B2 fue el más frecuente, con 36 % (n=21) de los casos, seguido de los grupos A o C y B1, con 24 % (n=14) y 17 % (n=10), respectivamente. El otro 23 % de los aislamientos se distribuyó entre los filogrupos A o E, F y A (figura 3). Aunque la PCR múltiple utilizada es capaz de diferenciar los seis filogrupos, se requirió la extensión de la reacción con dos iniciadores más para diferenciar los patrones de bandas similares entre los grupos A y C, y entre los D y E .

En el caso del filogrupo B2, el 57 % (n=12) de las cepas se aisló de muestras de orina, el 33 % (n=7) provenía del servicio de urología, y todas registraron resistencia a la ciprofloxacina (100 %). En el caso de las cepas pertenecientes a los filogrupos A o C, el 100 % (n=14) se aisló de sangre y orina, 50 % para cada tipo de muestra; el 72 % provenía de medicina interna y urología, con un 36 % para cada servicio, y una de las dos cepas resistentes a carbapenémicos pertenecía a este filogrupo.

De las cepas del filogrupo B1, el 60 % (n=6) se aisló de sangre; el 40 % (n=4) provenía de medicina interna y el 70 % era resistente a ciprofloxacino. Se encontró una asociación estadística (p<0,05) entre los grupos filogenéticos, la edad del paciente en que se había aislado la cepa y la resistencia a ciprofloxacino.

Los resultados completos de los grupos filogenéticos y las demás variables se presentan con mayor detalle en el cuadro 1. Para el caso de los genes de resistencia a betalactámicos, se encontró una asociación estadística marginal (p=0,058) entre las cepas CTX-M y la resistencia a ciprofloxacina (cuadro 2).

## Discusión

El estudio de la diversidad genética de los principales microorganismos causantes de infecciones hospitalarias en pacientes de cáncer, como *E. coli*, poco se ha abordada en nuestro medio, donde su frecuencia es alta ^5^. Mediante PCR múltiple para la detección de grupos filogenéticos de *E. coli,* en este estudio se evidenció la gran diversidad genética de los aislamientos de cepas productoras de BLEE obtenidos en pacientes con cáncer hospitalizados. Se detectaron, asimismo, gran resistencia a antibióticos no betalactámicos, como la quinolonas y los aminoglucósidos, y se determinaron los tipos de muestras de los aislamientos, los servicios de hospitalización de donde provenían y, por último, la relación de estas variables con los grupos filogenéticos.

Las producción de BLEE en aislamientos de *E. coli* es un problema mundial y muy frecuente en Latinoamérica ^12^^,^^13^. En este estudio, se demostró el importante predominio de la betalactamasa del *blaCTX-M* en *E. coli*, con un valor comparable al 93 % reportado en Nepal ^14^ y el 78 % en Sudán ^15^. Es importante mencionar que esta betalactamasa es la más diseminada en el mundo desde su aparición ^16^. A nivel regional, en Venezuela se ha reportado un 76,6 % de prevalencia ^17^, en México un 95% ^18^ y en Perú un 94,4 % en un hospital pediátrico ^19^. Debido a la naturaleza de la CTX-M, los carbapenémicos y los antibióticos no betalactámicos son las opciones más utilizadas en el tratamiento de las infecciones que causan, aunque la elección depende de otros factores como tipo de paciente, foco de infección, edad, comorbilidades, etc.

En este estudio, los aislamientos mostraron una gran resistencia a la gentamicina, mucho mayor que el ~ 30 % reportado en Nepal ^14^y comparable con el 58,3 % reportado en otro hospital de Perú ^19^, aunque menor del 82,2 % descrito en México ^18^. Es importante resaltar que, en este estudio, la resistencia a amikacina fue muy escasa (3 %) y se evidenció una importante resistencia a ciprofloxacina (91 %), valor que difiere de lo descrito en Yemen ^20^ donde se reportó 36,6 % de resistencia a quinolonas en cepas de *E. coli* productoras de BLEE del tipo CTX-M. Sin embargo, los resultados son similares a lo descrito en México, donde se reportó 91,1 % de resistencia en cepas BLEE, y en Chile, donde fue del 100 % ^18^^,^^21^.

Por otro lado, se evidenció el predominio de los filogrupos B2 y B1 en los servicios de hospitalización. Estos filogrupos pertenecen a *E. coli* patógenas extraintestinales, cuya distribución ya ha sido ampliamente descrita; por ejemplo, en Estados Unidos se ha reportado un 35,8 % del filogrupo B2 ^22^, lo que coincide con lo reportado en este estudio. Sin embargo, la frecuencia del grupo B1 en nuestro estudio difiere de la mayoría de los resultados publicados, ya que generalmente este se describe como grupo comensal ^23^. En un estudio en Venezuela, se reportan como grupos predominantes en ambientes hospitalarios el B2 (41 %), el A y el E (21 %) ^17^. Cabe mencionar que los aislamentos reportados como del grupo A o C en este estudio, probablemente pertenecen al filogrupo C, ya que el A incluye generalmente cepas comensales de *E. coli*^24^. Asimismo, se ha evidenciado que las cepas de *E. coli* del filogrupo B2 poseen mayor número de factores de virulencia y mayor resistencia, como se registró en este estudio al evaluar la relación entre los grupos filogenéticos y la resistencia a ciprofloxacino. En este filogrupo se encuentra el tipo ST131 de *E. coli*, ya descrito anteriormente ^25^.

Una de las principales limitaciones de nuestro estudio fue la imposibilidad de diferenciar el filogrupo A del C y el filogrupo D del E, pues no se utilizaron los iniciadores específicos en la segunda parte del protocolo descrito por Clermont, *et al*. ^6^, aunque ello no interfirió excesivamente con el desarrollo del estudio, ya que se identificó sin problemas el filogrupo B2, que era el de mayor interés. Otra limitación fue que las cepas solo se caraterizaron molecularmente para el gen _*bla*_ *CTX-M* y no para los otros genes de resistencia a betalactámicos, como el *TEM* o el *SHV*.

En conclusión, se evidenció una gran diversidad genética en los aislamientos de *E. coli* provenientes de muestras clínicas de pacientes con cáncer hospitalizados. Se observó un mayor predominio del filogrupo B2, así como una gran resistencia a antibióticos no betalactámicos, como las quinolonas y los aminoglucósidos. Asimismo, se registró una alta frecuencia de betalactamasas de tipo CTXM. La incorporación del estudio genético de las bacterias causantes de infecciones intrahospitalarias amplía la posibilidad de detectar brotes hospitalarios, y establecer la asociación con los perfiles y factores de resistencia de importancia epidemiológica como las BLEE.
